# The time horizon matters: results of an exploratory study varying the timeframe in time trade-off and standard gamble utility elicitation

**DOI:** 10.1007/s10198-015-0740-7

**Published:** 2015-11-26

**Authors:** Louis S. Matza, Kristina S. Boye, David H. Feeny, Lee Bowman, Joseph A. Johnston, Katie D. Stewart, Kelly McDaniel, Jessica Jordan

**Affiliations:** 1 Outcomes Research, Evidera, 7101 Wisconsin Avenue, Suite 1400, Bethesda, MD 20814 USA; 2 Eli Lilly and Company, Indianapolis, IN USA; 3 Department of Economics, McMaster University, Hamilton, ON Canada

**Keywords:** Utility, Time trade-off, Standard gamble, Time horizon, I1

## Abstract

**Introduction:**

The purpose of this study was to examine whether the time horizon of time trade-off (TTO) and standard gamble (SG) utility assessment influences utility scores and discrimination between health states.

**Methods:**

In two phases, UK general population participants rated three osteoarthritis health states in TTO and SG procedures with two time horizons: (1) 10-year and (2) a time horizon derived from self-reported additional life expectancy (ALE). The two time horizons were compared in terms of mean utilities and discrimination among health states.

**Results:**

In Phase 1, the 10-year tasks were completed by 80 participants, 35 of whom also completed utility assessment with the ALE. In Phase 2, all 101 participants completed procedures with both time horizons. Utility scores tended to be lower with the ALE than the 10-year, a difference that was statistically significant for two health states with SG in Phase 1 (*P* < 0.05), two health states with TTO in Phase 2 (*P* < 0.01), and one health state with SG in Phase 2 (*P* < 0.001). In Phase 1, rates of discrimination between mild and moderate osteoarthritis health states were significantly higher with the ALE than the 10-year (TTO: *P* = 0.03; SG: *P* = 0.001). This pattern of discrimination was similar in Phase 2.

**Discussion:**

Results suggest that the time horizon could influence utility scores and discrimination among health states. When designing utility evaluations, researchers should carefully consider the time horizon so that the value of health states is accurately represented in cost-utility models.

## Introduction

The time trade-off (TTO) technique may be the most common approach for obtaining health state utilities for use in cost-utility analyses of medical treatments. The TTO method involves a choice between living in a particular health state for a given period of time or living in full health for a shorter period of time [1, 2]. The amount of time in full health is varied until the respondent is indifferent between the two alternatives. Based on the point at which the respondent is indifferent between the two alternatives, the health state is assigned a utility score, anchored to values of 0 representing dead and 1 representing full health.

Because the TTO procedure depends on perceptions of time and life expectancy, the duration of time spent in the health state is a key component of the task. This time horizon varies across studies, and researchers must choose a time horizon when designing a TTO utility assessment. The most common TTO time horizon appears to be 10 years [3–10]. This time horizon was used in the influential Measurement and Valuation of Health (MVH) study, which identified utilities of EQ-5D health states in a representative sample of the UK population [11, 12]. It is likely that many researchers use this time horizon to maximize consistency with the EQ-5D, which is the utility assessment method preferred by the National Institute for Health and Care Excellence (NICE) [13]. Although most published TTO studies do not report justification for the time horizon, some articles have stated that the 10-year time horizon was selected to be consistent with the MVH methods [14, 15].

A wide range of other TTO time horizons have also been used [16], including 2 years [17, 18], 5 years [19, 20], 16 years [21], 20 years [22–25], 30 years [26–28], 36 years [29], and 50 years [30]. As an alternative approach, some studies have used an additional life expectancy (ALE) time horizon for each respondent, depending on the respondent’s age and ALE [31–38]. In contrast to the studies using a fixed time horizon for all respondents, this ALE approach aims to present respondents with realistic choices that correspond to reasonable expectations for their own lifespan without introducing bias that can stem from a fixed time horizon perceived as either a gain or a loss [39].

Regardless of the TTO time horizon, the resulting utility is interpreted on the same scale anchored to values of 0 representing dead and 1 representing full health, and utilities from studies with different time horizons are generally considered to be comparable when used in cost-utility models. However, previous studies suggest that the time horizon may influence the utility value [40]. Two of these studies found that patients tend to trade a greater proportion of time when the TTO task was conducted with a longer time horizon, resulting in lower mean utility scores [41, 42]. However, because each respondent rated only a single health state (i.e., his/her own), the results do not illustrate the potential impact of TTO time horizon on utility differences between multiple health states. The distinction among multiple health states is a key outcome of many health state utility studies, and these differences can have a substantial impact on the results of a cost-utility model that uses the utility scores.

Therefore, the primary purpose of the current study was to examine whether the TTO time horizon influences the resulting utility scores and discrimination among health states. Respondents rated multiple hypothetical health states (often called “vignettes”) in TTO interviews with two of the most commonly used time horizons: a 10-year time horizon and an ALE time horizon derived from each respondent’s self-reported additional life expectancy.

A secondary goal was to examine the influence of time horizon on the results of standard gamble (SG) utility assessment. SG is often referred to as the “classical” utility valuation method because it is grounded in expected utility theory [1, 43, 44]. When valuing health states with the SG method, respondents are given a choice between certainty (i.e., living in the health state being rated) and uncertainty. The uncertain option is presented in the form of a gamble between two possible outcomes including one that is more preferable than the health state being rated (e.g., full health) and another that is less preferable than the health state being rated (e.g., dead). Because SG procedures focus on probabilities as well as time, the choice of time horizon might have a less direct impact on SG than on TTO. Still, when completing an SG task, respondents are told how much time will be spent in the health states, and this time horizon could influence the results.

## Methods

### Overview of study design

This study was conducted in two phases. Phase 1 was conducted as part of a larger study that included a one-on-one utility interview in which all participants first rated three health-state descriptions in TTO and SG tasks with a 10-year time horizon. Then, participants were randomized to rate the health states again in one of two variations of the TTO and SG tasks. Half of the sample was randomized to a group that performed the TTO and SG tasks again, but with an ALE time horizon instead of the 10-year time horizon. The other half performed a different task that was not included in the current analysis. Therefore, in Phase 1, the sample for the 10-year time horizon was roughly twice as large as the sub-group who completed the tasks with the ALE time horizon.

The results of Phase 1 suggested that the time horizon may have an impact on results. Therefore, Phase 2 was conducted to replicate the time horizon comparison and examine whether a similar pattern of results emerged. All participants rated the health states in TTO and SG tasks with both the 10-year time and ALE time horizons. In Phase 2, the order of TTO versus SG and the order of 10-year versus ALE were randomized.

### Participants

The current study was conducted with general population respondents in accordance with recommendations from reimbursement agencies suggesting that utilities should represent general population preferences [13, 45, 46]. Participants in both phases were required to be at least 18 years old, reside in the United Kingdom, and be able to give written informed consent. Participants were not eligible if they had cognitive impairment, hearing difficulty, visual impairment, severe psychopathology, or insufficient knowledge of English that could interfere with the ability to complete study measures. Study inclusion criteria did not require any particular clinical characteristics. Phase 2 had two additional exclusion criteria: individuals were excluded (1) if they participated in Phase 1; or (2) if their self-reported ALE was less than 15 years.

Participants were recruited through newspaper and online advertisements. For Phase 1, a total of 197 potential participants responded to the newspaper advertisements, and 109 of these were reached for screening to assess whether they met study inclusion criteria. Of the 109 screened participants, 2 were ineligible, 101 were scheduled for interviews, and 81 attended interviews. Of the 81 participants, 1 was unable to complete any of the utility procedures, while 2 others were unable to complete the SG. Therefore, the sample includes 80 participants with TTO data and 78 with SG data. Of the 80 participants, 39 were randomized to perform the tasks with the ALE time horizon.

For Phase 2, a total of 274 potential participants responded by telephone or email to the newspaper or online advertisements, and 174 of these were reached for screening. Of the 174 screened participants, 148 were eligible, 141 were scheduled for interviews, and 108 attended the interviews. All participants were rescreened at the time of the interview, and 4 of the 108 participants were found to be ineligible for the following reasons: unable to read and write English, previously participated in Phase 1, self-reported ALE less than 15 years, and unwilling to complete the study demographic form. Three of the 104 eligible participants were unable to complete the utility interview procedures to provide valid TTO and SG data. Thus, a total of 101 valid utility interviews were conducted. All analyses were performed with the sample of participants who provided valid TTO and SG data (*n* = 101).

### Health states

Three osteoarthritis health states associated with elective total hip arthroplasty were presented during the utility interview (see Appendix A for health state texts). These health states were based on health states used in two previous studies describing patients with mild (health state A), moderate (B), or severe (C) osteoarthrosis of the hip [47, 48], with minor edits made so that they would be appropriate for administration in the UK. These health states were selected for use in the current study because they were brief and easy to understand, with clear differences among them.

### Utility interview procedures and scoring

Individual interviews were conducted in London during November 2011 (Phase 1) and May–June 2013 (Phase 2). All procedures were approved by an independent Institutional Review Board, and participants provided written informed consent prior to completing any parts of the study. The health states and procedures were presented following a standardized interview script.

First, participants rated the health states using a visual analogue scale (VAS) intended to introduce them to the health states. Health states were presented on individual cards, and the VAS had anchors of 0 (dead) and 100 (full health). Then, health state utilities were obtained using TTO and SG methods [1, 43]. In Phase 1, the TTO and SG tasks were first administered with the 10-year time horizon. The order of TTO and SG was randomized. After completing the tasks with the 10-year time horizon, half of the participants were randomized to complete the tasks with the ALE time horizon. In Phase 2, all participants completed the TTO and SG tasks with both the 10-year and ALE time horizons. The order of the time horizons and the utility assessment method (i.e., TTO and SG) was randomized.

In the 10-year TTO task, participants were offered a choice between spending 10 years in the health state being rated versus spending varying shorter amounts of time in full health, followed by death. Time was varied in 1-year increments. The utility was calculated based on the choice in which the respondent was indifferent between *y* years in the health state being evaluated (i.e., 10 years) and *x* years in full health (followed by *y−x* years dead). The resulting utility (*u*) is calculated as *u* = *x*/*y*.

In the 10-year SG task, participants were offered a choice between two alternatives, one that was certain and one that was uncertain. Choice A was the uncertain choice with two potential outcomes: either to remain in full health for the 10-year period with a probability of *P* or death with a probability of 1 − *P*. Choice B was to remain in the health state being rated for 10 years. Probability *P* was varied in 10 % increments until the participant was indifferent between choices A and B, and the resulting utility is equal to *P* at this point of indifference.

For the ALE time horizon, participants were first asked how many additional years they expected to live. Then, they completed the TTO and SG tasks with time horizons of either 20, 30, 40, or 50 years, depending on which time horizon corresponded most closely to their self-reported additional life expectancy. Participants who reported an additional life expectancy of less than 15 years did not complete the tasks with the ALE time horizon because their additional life expectancy did not differ substantially from the 10-year time horizon.

Increments in the TTO task with the ALE time horizon were proportional to those in the 10-year tasks described above. For example, participants completing a TTO task with a 40-year time horizon were presented with choices varying by increments of 4 years. Because the SG choices vary by percentages rather than years, the task with the ALE time horizon was the same as the task with the 10-year time horizon, except participants were told that they would be living in the health states for a longer period of time (i.e., 20–50 years, depending on self-reported additional life expectancy).

### Statistical analysis procedures

Continuous variables including utilities were summarized in terms of means and standard deviations. Categorical variables were summarized as frequencies and percentages. Comparisons between two pairs of health states were examined in the current analysis: A versus B and B versus C. For each of these two pairs, mean utility values are presented for all four utility assessment methods: 10-year TTO, 10-year SG, TTO with the ALE time horizon, and SG with the ALE time horizon. The primary questions of the current study involved the extent to which the four methods differed in terms of mean health state utilities and the percentage of respondents who discriminated between health state pairs. The frequency and percentage of respondents who discriminated between each health state pair is presented for each of the four methods. To examine whether the rate of discrimination significantly differed depending on the time horizon, follow-up Chi square analyses were conducted for key comparisons.

## Results

### Sample description

The Phase 1 sample included 80 participants with a mean age of 47.3 years (SD = 14.4) and were 48.8 % female (Table 1). Most participants reported their ethnicity as white (62.5 %). The majority of the sample was employed either full time (25.0 %) or part time (35.0 %). The Phase 2 sample included 101 participants who had a mean age of 37.8 years (SD = 14.4) and were 49.5 % female (Table 1). As in Phase 1, the majority of participants reported their ethnic/racial background as white (60.4 %) and were employed either full time (31.7 %) or part time (28.7 %). In both phases, there were some respondents who reported having arthritis [7 (8.8 %) of the 80 respondents in Phase 1; 2 (2.0 %) of the 101 respondents in Phase 2], which could be similar to the condition described in the health states.

**Table 1:** Demographic characteristics

Demographic characteristics	Phase 1	Phase 2
Age (mean, SD)	47.3 (14.4)	37.8 (14.4)
Gender (*n*, %)		
Male	41 (51.3 %)	51 (50.5 %)
Female	39 (48.8 %)	50 (49.5 %)
Ethnicity (*n*, %)		
White	50 (62.5 %)	61 (60.4 %)
Black	13 (16.3 %)	12 (11.9 %)
Asian	8 (10.0 %)	20 (19.8 %)
Other	9 (11.3 %)	8 (7.9 %)
Marital status (*n*, %)		
Single	37 (46.3 %)	67 (66.3 %)
Married/living with partner	23 (28.8 %)	22 (21.8 %)
Divorced/separated/widowed	20 (25.0 %)	12 (11.9 %)
Employment status (*n*, %)		
Full-time work	20 (25.0 %)	32 (31.7 %)
Part-time work	28 (35.0 %)	29 (28.7 %)
Unemployed	11 (13.8 %)	12 (11.9 %)
Other^a, b^	21 (26.3 %)	28 (27.7 %)
Education level (*n*, %)		
No university degree^c, d^	41 (51.3 %)	41 (40.6 %)
University or postgraduate degree	39 (48.8 %)	60 (59.4 %)

^a^Phase 1: Other employment includes homemaker (*n* = 3), student (*n* = 2), retired (*n* = 15), and disabled (*n* = 1)

^b^Phase 2: Other employment includes homemaker (*n* = 2), student (*n* = 15), retired (*n* = 8), disabled (*n* = 1), and other (*n* = 2)

^c^Phase 1: No university degree includes no formal qualifications (*n* = 1), GCSE/O levels (*n* = 17), A levels (*n* = 10), and vocational/work based qualifications (*n* = 13)

^d^Phase 2: No university degree includes no formal qualifications (*n* = 3), GCSE/O levels (*n* = 12), A levels (*n* = 14), vocational/work based qualifications (*n* = 11), and college (*n* = 1)

### Self-reported ALE

At the beginning of the ALE time horizon task, participants were asked to estimate their ALE. Their responses were rounded to the nearest 10-year milestone so they could be categorized into time horizon groups for the utility assessment procedures (Table 2). In Phase 1, 39 participants were randomized to complete the utility tasks with the ALE time horizon. Four (10.3 %) of these participants reported additional life expectancy of less than 15 years, which rounded to 10 years. Therefore, they were not asked to complete utility assessments with the ALE time horizon.

**Table 2:** Frequency of participants in additional life expectancy (ALE) categories

Life expectancy categories^a^	ALE time horizon				Respondent’s age	ALE + age
	*N*	Mean (SD)	Minimum	Maximum	Mean (SD)	Mean (SD)
Phase 1						
0–14 years	4 (10.3 %)	10.3 (1.3)	9	12	60.5 (10.2)	70.8 (10.6)
15–24 years	7 (17.9 %)	19.6 (2.1)	15	22	59.7 (8.9)	79.3 (8.0)
25–34 years	8 (20.5 %)	29.3 (3.0)	25	34	47.5 (6.9)	76.8 (6.5)
35–44 years	6 (15.4 %)	39.8 (2.9)	35	44	37.3 (11.7)	77.2 (11.9)
45 years or more	14 (35.9 %)	55.6 (10.1)	45	80	31.9 (10.3)	87.4 (10.0)
Phase 2						
15–24 years	13 (12.9 %)	18.8 (2.6)	15	23	54.2 (16.9)	73.1 (16.8)
25–34 years	20 (19.8 %)	28.9 (2.8)	25	34	49.4 (11.7)	78.3 (11.9)
35–44 years	20 (19.8 %)	38.7 (2.5)	35	43	39.1 (10.1)	77.7 (10.2)
45 years or more	48 (47.5 %)	57.5 (9.0)	45	80	27.9 (6.3)	85.4 (8.1)

^a^ In Phase 1, this question was presented only to the subgroup of 39 respondents who were randomized to complete utility tasks with the ALE time horizon. The 4 respondents in the 0–14 year category did not complete the ALE time horizon tasks because their self-reported ALE rounded to 10 years. In Phase 2, all participants responded to this question

The other 35 participants in Phase 1 reported that they expected to live more than 14 additional years, and they completed utility procedures with the ALE time horizon that most closely matched their additional life expectancy. As shown in Table 2, seven participants (17.9 %) reported additional life expectancy within the range of 15–24 years, and therefore completed the utility tasks with a 20-year time horizon. Other participants were categorized in the 30-year group (*n* = 8; 20.5 %), 40-year group (*n* = 6; 15.4 %), or the 50-year group (*n* = 14; 35.9 %).

In Phase 2, potential participants were excluded from the study if they expected to live fewer than 15 years. As in Phase 1, participants were categorized into time horizon groups that most closely matched their self-reported ALE. The four time horizon groups were as follows: 20-year (*n* = 13; 12.9 %), 30-year (*n* = 20; 19.8 %), 40-year (*n* = 20; 19.8 %), and 50-year (*n* = 48; 47.5 %).

### Mean utilities

Table 3 presents mean health state utilities in Phase 1 and Phase 2. In Phase 1, health state A had a mean TTO utility score of 0.85 with both the 10-year and ALE time horizons. However, TTO utilities for health states B and C and SG utilities for all three health states were consistently lower with the ALE time horizon than with the 10-year time horizon. These differences between the two time horizons ranged from 0.03 to 0.09, and the difference was statistically significant for health states A and B in the standard gamble (A: 0.86 versus 0.89; *P* = 0.02 and B: 0.76 versus 0.85; *P* = 0.0067).

**Table 3:** Mean utilities

Health state	*N* ^a^	10-year time horizon mean (SD)	ALE time horizon mean (SD)	Difference between the two time horizons mean (SD)	*t* statistic	*P* value
**Phase 1**						
TTO						
A (mild)	35	0.85 (0.14)	0.85 (0.14)	−0.00 (0.14)	−0.1	0.95
B (moderate)	34	0.76 (0.18)	0.74 (0.20)	0.03 (0.16)	1.0	0.34
C (severe)	26	0.50 (0.31)	0.46 (0.28)	0.06 (0.21)	1.5	0.15
SG						
A (mild)	35	0.89 (0.13)	0.86 (0.13)	0.03 (0.07)	2.4	0.02
B (moderate)	34	0.85 (0.15)	0.76 (0.21)	0.09 (0.18)	2.9	0.0067
C (severe)	26	0.60 (0.35)	0.58 (0.33)	0.03 (0.23)	0.7	0.47
**Phase 2**						
TTO						
A (mild)	101	0.84 (0.14)	0.82 (0.16)	0.02 (0.15)	1.6	0.12
B (moderate)	101	0.74 (0.24)	0.68 (0.26)	0.05 (0.17)	3.2	0.0019
C (severe)	101	0.39 (0.45)	0.33 (0.48)	0.06 (0.19)	2.9	0.0047
SG						
A (mild)	101	0.86 (0.15)	0.85 (0.15)	0.01 (0.15)	1.0	0.34
B (moderate)	101	0.77 (0.26)	0.75 (0.25)	0.02 (0.18)	1.4	0.17
C (severe)	101	0.44 (0.44)	0.36 (0.44)	0.08 (0.21)	3.8	0.0002

*ALE* additional life expectancy time horizon, *SD* standard deviation, *SG* standard gamble, *TTO* time trade-off

^a^ *N* is the number of respondents who completed tasks with both time horizons for each health state. In Phase 1, *N* for health states B and C varies because participants who rated a health state as negative (i.e., worse than dead) were not given a utility score. In Phase 2, if participants indicated that a health state was worse than dead, they were offered a choice between immediate death (alternative 1) and a life span (alternative 2) beginning with varying amounts of time in the health state being rated, followed by full health. For these negative utilities, the current study used a common scoring approach that limits the score range between 0 and −1 (formula: *u* = −*x*/*t*, where *x* is time in full health, and *t* is the total life span of alternative 2 in the TTO choice) [1, 12, 43]

In Phase 2, all health state utilities were lower with the ALE time horizon than with the 10-year time horizon. The utility difference between the two time horizons ranged from 0.02 to 0.06 in the TTO, with a statistically significant difference between time horizons for health states B and C (B: 0.68 versus 0.74; *P* = 0.0019 and C: 0.33 versus 0.39; *P* = 0.0047). In the SG, the utility difference between the two time horizons ranged from 0.01 to 0.08, with a statistically significant difference between time horizons for health state C (0.36 versus 0.44; *P* = 0.0002).

### Differentiating between health states A and B: Phase 1

With all assessment methods, health state A representing mild osteoarthritis had a higher mean score than health state B representing moderate osteoarthritis (Table 4). In Phase 1, the mean difference between the two health states was 18 on the VAS with a possible range of 0–100. Utility differences between the two health states were 0.06 with the 10-year SG, 0.09 with the 10-year TTO, and 0.11 with the ALE time horizons. Nearly all participants (97.5 %) rated health state A higher than health state B on the VAS, indicating that participants could distinguish between these two health states, and there was a consistent preference for A over B. However, the 10-year tasks did not detect this preference for the majority of respondents. In the 10-year TTO task, only 32.5 % of respondents distinguished between A and B. In the 10-year SG task, only 23.1 % of respondents distinguished between these two health states. In TTO and SG tasks with the ALE time horizon, 54.3 % of participants differentiated between health states A and B, suggesting that the ALE time horizon resulted in greater discrimination between health states than the 10-year time horizon.

**Table 4:** Comparison between health states A (mild osteoarthritis) and B (moderate osteoarthritis)

Utility assessment method	*N* ^a^	Mean utility values		Difference between health states A and B	Frequency and percentage of respondents differentiating between health states A and B (i.e., utility of A > utility of B)
		A (mild)	B (moderate)		
Phase 1					
VAS	80	75.20	57.18	18.03	78 (97.5 %)
TTO 10-year	80	0.88	0.79	0.09	26 (32.5 %)
TTO ALE	35	0.85	0.74	0.11	19 (54.3 %)
SG 10-year	78	0.91	0.86	0.06	18 (23.1 %)
SG ALE	35	0.86	0.76	0.11	19 (54.3 %)
Phase 2					
VAS	101	75.93	51.14	24.79	101 (100.0 %)
TTO 10-year	101	0.84	0.74	0.11	52 (51.5 %)
TTO ALE	101	0.82	0.68	0.14	63 (62.4 %)
SG 10-year	101	0.86	0.77	0.09	35 (34.7 %)
SG ALE	101	0.85	0.75	0.10	46 (45.5 %)

*ALE* additional life expectancy time horizon, *SG* standard gamble, *TTO* time trade-off, *VAS* visual analog scale

^a^Number of respondents who participated in each utility assessment method

Chi square analyses were conducted to examine whether there was a statistically significant difference between the 10-year and ALE time horizons with regard to the frequency of participants who differentiated between health states A and B (Table 5). For the TTO methods, results of the 2 by 2 Chi square indicate that there was a significant difference between the two time horizons in terms of health state differentiation (*χ*^2^ = 4.9; *P* = 0.03). Results followed the same pattern for the SG methods (*χ*^2^ = 10.7; *P* = 0.001).

**Table 5:** Health state A (mild osteoarthritis) versus health state B (moderate osteoarthritis): Chi square analysis comparing 10-year time horizon to additional life expectancy (ALE) time horizon

Health state preferences (*n*, %)	Time horizon in each utility task		Chi square	*P* value
	10-year time horizon	ALE time horizon^a^		
**Phase 1**				
TTO				
A > B^b^	26 (32.5 %)	19 (54.3 %)	4.9	0.03
A = B^c^	54 (67.5 %)	16 (45.7 %)		
SG				
A > B^b^	18 (23.1 %)	19 (54.3 %)	10.7	0.001
A = B^c^	60 (76.9 %)	16 (45.7 %)		
**Phase 2**				
TTO				
A > B^b^	52 (51.5 %)	63 (62.4 %)	2.4	0.12
A = B^c^	49 (48.5 %)	38 (37.6 %)		
SG				
A > B^b^	35 (34.7 %)	46 (45.5 %)	2.5	0.11
A = B^c^	66 (65.3 %)	55 (54.5 %)		

*ALE* additional life expectancy time horizon, *SG* standard gamble, *TTO* time trade-off

^a^The ALE time horizon was 20, 30, 40, or 50 years, depending on the self-reported additional life expectancy of each participant

^b^This row includes the frequency and percentage of participants who provided a greater utility value for health state A than for health state B

^c^This row includes the frequency and percentage of participants who provided the same utility value for health state A and health state B

### Differentiating between health states A and B: Phase 2

In Phase 2, the mean difference between health states A and B was 24.8 with the VAS, 0.09 with the 10-year SG, 0.11 with the 10-year TTO, 0.10 with the ALE SG, and 0.14 with the ALE TTO. In the 10-year TTO task, 51.5 % of respondents distinguished between A and B, compared with 62.4 % in the ALE TTO task. In the 10-year SG task, 34.7 % of respondents distinguished between these two health states, compared with 45.5 % in the ALE SG task. Although these rates of differentiation were numerically higher with the ALE time horizon than the 10-year time horizon, Chi square analyses did not find a significant difference between the time horizons (*χ*^2^ = 2.4; *P* = 0.12 for TTO and 2.5; *P* = 0.11 for SG).

### Differentiating between health states B and C: Phase 1

With all assessment methods, health state B representing moderate osteoarthritis had a higher mean score than health state C representing severe osteoarthritis (Table 6). In Phase 1, the mean difference between the two health states was 27 on the VAS, 0.21 with both SG methods, 0.25 with the 10-year TTO, and 0.31 with the ALE time horizon TTO.

**Table 6:** Comparison of responses between health states B (moderate osteoarthritis) and C (severe osteoarthritis)

Utility assessment method	*N* ^a^	Mean utility values		Difference between health states B and C	Frequency and percentage of respondents differentiating between health states B and C (i.e., utility of B > utility of C)
		B (moderate)	C (severe)		
Phase 1					
VAS	80	57.18	30.92	26.59	79 (98.8 %)
TTO 10-year	80	0.79	0.59	0.25	62 (77.5 %)
TTO ALE	35	0.74	0.46	0.31	30 (85.7 %)
SG 10-year	78	0.86	0.68	0.21	56 (71.8 %)
SG ALE	35	0.76	0.58	0.21	24 (68.6 %)
Phase 2					
VAS	101	51.14	30.11	21.03	101 (100.0 %)
TTO 10-year	101	0.74	0.39	0.35	89 (88.1 %)
TTO ALE	101	0.68	0.33	0.35	92 (91.1 %)
SG 10-year	101	0.77	0.44	0.33	83 (82.2 %)
SG ALE	101	0.75	0.36	0.38	91 (90.1 %)

*ALE* additional life expectancy time horizon, *SG* standard gamble, *TTO* time trade-off, *VAS* visual analog scale

^a^Number of respondents who participated in each utility assessment method

With all utility methods in Phase 1, the majority of respondents differentiated between these two health states. All but one of the 80 participants (98.8 %) rated health state B higher than health state C on the VAS, and this difference between the two health states was also reflected in the TTO and SG utility tasks for most participants (Table 6). Chi square analyses examining rates of differentiation between B and C did not yield statistically significant differences between the 10-year and ALE time horizons (TTO: *χ*^2^ = 1.0, *P* = 0.31; SG: *χ*^2^ = 0.12, *P* = 0.73). Although the difference between the two time horizons was not statistically significant, the TTO with an ALE time horizon did result in a slightly greater rate of differentiation than the TTO with a 10-year time horizon (85.7 versus 77.5 %).

### Differentiating between health states B and C: Phase 2

In Phase 2, the mean difference between the two health states was 21 on the VAS, 0.35 with both TTO methods, 0.33 with the 10-year SG, and 0.38 with the ALE time horizon SG. With all utility methods, the majority of respondents differentiated between these two health states. All participants (100.0 %) rated health state B higher than health state C on the VAS, and this difference between the two health states was also reflected in the TTO and SG utility tasks for most participants (Table 6). Although a slightly higher rate of respondents differentiated between health states with the ALE time horizon than the 10-year time horizon (91.1 versus 88.1 % with TTO; 90.1 versus 82.2 % with SG), Chi square analyses did not find statistically significant differences between the two time horizons (TTO: *χ*^2^ = 0.48, *P* = 0.49; SG: *χ*^2^ = 2.65, *P* = 0.10).

## Discussion

Findings add to previous literature suggesting that the time horizon of the utility assessment task could have an impact on the results [10, 41, 42, 49]. In the current analyses comparing the 10-year time horizon to the ALE time horizon, two trends emerged. First, the longer time horizon appears to lead to increased rates of discrimination among health states, which has not been examined in previous research. The difference between the two time horizons emerged primarily in comparisons between health states A (mild osteoarthritis) and B (moderate osteoarthritis). Compared with the 10-year time horizon, the ALE approach had a greater ability to detect and quantify respondents’ preferences for health state A over health state B. This finding highlights the importance of the time horizon by showing that it may influence the extent to which direct utility elicitation can distinguish among health states. Essentially, TTO studies using the 10-year time horizon to be consistent with the MVH study could fail to detect some meaningful differences between health states.

When assessing utilities of multiple health states, it is essential that the assessment method be able to detect meaningful differences in preference. If one health state is truly preferred over another, the resulting utilities must reflect this distinction so that the difference between health states can be represented accurately in a cost-utility analysis. Distinctions among health state utilities have a direct impact on the results of cost-utility models, and failure to detect true differences in health state preference would limit the accuracy and usefulness of any model using the utilities. Because healthcare policy and reimbursement decisions are often directly informed by the results of cost-utility models, accurate distinction between health states is critical.

The difference between the time horizons was not as pronounced or consistent when examining discrimination between health states B and C. The great majority of respondents had a clear preference for B over C regardless of the utility assessment method. It seems likely that the utility assessment approach has less influence on discrimination when differences between health states are more substantial because these differences will be detected with almost any assessment method.

The second trend is that utility scores tended to be lower with the ALE time horizon than with the 10-year time horizon (Table 3). This result is consistent with previous studies reporting that longer TTO time horizons yielded more willingness to trade, resulting in lower utility scores, among respondents rating their own health [41, 42]. The current study adds to these previous results by demonstrating that a longer TTO time horizon was associated with lower utility scores when rating hypothetical health states instead of one’s own health. In addition, while previous studies have focused on the TTO time horizon [40], current results suggest that the time horizon may also influence results in SG utility assessment.

It should be noted that the utility differences between time horizons varied across the health states and methods. The ALE time horizon resulted in lower utility scores than the 10-year time horizon in 11 of the 12 comparisons presented in Table 3, with the magnitude of mean differences ranging from 0.01 to 0.09. The magnitude of these differences exceeded a suggested guideline for a clinically important difference (at least 0.05) [50] in two of six comparisons in Phase 1 and three of six in Phase 2. Although this difference was small in some cases, small differences between health states can often shift the outcome of a cost-utility model, particularly when modeling a large number of patients over a long timespan. Therefore, the choice of time horizon in a valuation study could have a substantial influence on the outcome of a model using the resulting utility values as inputs.

Although this study was not designed to test the QALY (i.e., quality-adjusted life year) model, results add to literature raising questions about the assumptions underlying the QALY. One key assumption, often referred to as *constant proportional trade*-*off*, is that “health state values must be independent of the duration of states [1].” Current results add to previous literature suggesting that duration can affect one’s valuation of a health state, which violates this assumption [40, 51].

The findings reported here should be interpreted with caution because, despite consistent trends across the two study phases, differences between the 10-year and ALE time horizons were not always statistically significant. For example, while the ALE time horizon was associated with lower utility scores than the 10-year time horizon in 11 of 12 comparisons (Table 3), only 5 of these 11 were statistically significant. In addition, although the ALE time horizon had greater rates of discrimination between health states A and B in both phases, this difference was only statistically significant in Phase 1. Perhaps the inconsistent statistical significance is related to the relatively small sample size, which is a study limitation. This was an exploratory study with limited resources designed to identify potential methodological issues. It is possible that the sample size did not offer sufficient statistical power to detect differences in all analyses. Therefore, while the current results generally support the hypothesis that the time horizon has an impact on utility scores and discrimination among health states, future research with larger samples is needed to provide confidence in this finding.

Another limitation is that this study was conducted with a small number of health states. It is possible that the impact of time horizon could be different for health states representing different types of medical or psychiatric conditions. Therefore, it is not known whether current findings are generalizable to other health states. Future research may examine the impact of time horizon across a wider range of disease characteristics and symptom severity.

Despite these limitations, given that the time horizon of a utility task could influence the results, researchers should carefully consider the advantages and disadvantages of possible time horizons when designing a direct utility elicitation study. The 10-year time horizon has two advantages. First, it is consistent with the commonly cited MVH valuation of EQ-5D health states, and a common assumption is that utilities derived from a 10-year TTO task may be comparable to EQ-5D utilities. Second, the 10-year time horizon simplifies interview procedures for interviewers and respondents.

However, the 10-year time horizon may not always be the most effective approach for identifying differences among health state preferences, particularly for younger respondents whose subjective ALE far exceeds 10 years [10]. The ALE approach has the advantage of presenting more realistic choices for each respondent. Because the task is consistent with reasonable expectations for each respondent’s own lifespan, the respondent can focus on the health state without the distraction of an unrealistic time horizon. In the current study, the ALE time horizon allowed for a more accurate utility assessment, as indicated by increased discrimination between mild and moderate osteoarthritis health states. Furthermore, the ALE approach does seem feasible as participants generally reported ALEs within a reasonable range, and greater ALE tended to be associated with younger current age (Table 2). Although no time horizon is likely to be optimal in all utility studies, current results suggest that researchers should think carefully about the choice of a time horizon, rather than assuming that a 10-year time horizon is always appropriate. The optimal time horizon for an individual study may be selected based on clinical characteristics of the health states, expected age of the target sample, and intended use of the utility values. In some situations, it may be beneficial to sacrifice comparability with other studies using the 10-year time horizon in favor of greater discrimination among health states and relevance to the context of a particular disease [52].

Regardless of which time horizon is used, it should be clearly stated and justified in publications because it could be a factor that influences results. Most articles reporting TTO studies mention the time horizon, but few provide justification for selecting a particular time horizon. Among articles providing justification, some reported choosing a time horizon to be consistent with the ALE associated with the medical condition represented in the health states [7, 19, 25, 30]. Others selected a 10-year time horizon to be consistent with MVH study methodology [14, 15].

In sum, results suggest that the time horizon of the utility assessment procedure could influence utility scores and the degree to which respondents distinguish among health states. Based on these findings, the time horizon merits further investigation in future research with larger samples and a wider range of health states. Furthermore, it is recommended that researchers carefully consider the selection of a time horizon when designing a TTO or SG utility study and provide justification for the selected time horizon when reporting study methods.

## Appendix A. Health states administered in this study

Health State A: Mild osteoarthritis associated with elective total hip arthroplasty.

1. You have slight hip pain and stiffness.
2. You don’t need a walking frame (Zimmer frame) or walking stick, but you sometimes use a piece of furniture to steady yourself.
3. You occasionally need to use over-the-counter pain medication (such as paracetamol, aspirin, or ibuprofen).
4. You have no night pain and you sleep well.
5. You are able to do all housework and chores if you take your time.
6. Your social activity with family and friends is only slightly decreased.

Health State B: Moderate osteoarthritis associated with elective total hip arthroplasty.

1. You have moderate pain and stiffness upon exertion.
2. You need to use a walking stick to walk more than a quarter of a mile (about 400 m).
3. You occasionally need to take prescription pain medication.
4. You sometimes experience night pain that is relieved by a position change and/or pain pills.
5. You can only do light housework or chores.
6. You socialise with family and friends, but more than 1 h is painful and tiring.

Health State C: Severe osteoarthritis associated with elective total hip arthroplasty.

1. You have almost constant pain and stiffness.
2. You must use a walking frame (Zimmer frame).
3. You regularly use prescription pain medication.
4. You sleep poorly at night.
5. You are unable to do most housework and/or chores.
6. You socialise with family and friends, but more than 30 min is painful and tiring.

## References

[1] Brazier J, Ratcliffe J, Salomon JA, Tsuchiya A (2007). Measuring and valuing health benefits for economic evaluation.

[2] Torrance GW (1976). Social preferences for health states: an empirical evaluation of three measurement techniques. Socio-econ. Plan. Sci..

[3] Ekman M, Berg J, Wimo A, Jonsson L, McBurney C (2007). Health utilities in mild cognitive impairment and dementia: a population study in Sweden. Int. J. Geriatr. Psychiatry.

[4] Joyce VR, Barnett PG, Bayoumi AM, Griffin SC, Kyriakides TC, Yu W, Sundaram V, Holodniy M, Brown ST, Cameron W (2009). Health-related quality of life in a randomized trial of antiretroviral therapy for advanced HIV disease. J. Acquir. Immune Defic. Syndr..

[5] Lloyd A, Hodgkins P, Sasane R, Akehurst R, Sonuga-Barke EJ, Fitzgerald P, Nixon A, Erder H, Brazier J (2011). Estimation of utilities in attention-deficit hyperactivity disorder for economic evaluations. Patient.

[6] Melsop KA, Boothroyd DB, Hlatky MA (2003). Quality of life and time trade-off utility measures in patients with coronary artery disease. Am. Heart J..

[7] Shauver MJ, Clapham PJ, Chung KC (2011). An economic analysis of outcomes and complications of treating distal radius fractures in the elderly. J. Hand Surg. Am..

[8] Sherman KE, Sherman SN, Chenier T, Tsevat J (2004). Health values of patients with chronic hepatitis C infection. Arch. Intern. Med..

[9] Swinburn P, Lloyd A, Nathan P, Choueiri TK, Cella D, Neary MP (2010). Elicitation of health state utilities in metastatic renal cell carcinoma. Curr. Med. Res. Opin..

[10] van Nooten FE, Koolman X, Brouwer WB (2009). The influence of subjective life expectancy on health state valuations using a 10 year TTO. Health Econ..

[11] Dolan P (1997). Modeling valuations for EuroQol health states. Med. Care.

[12] Dolan P, Gudex C, Kind P, Williams A (1996). The time trade-off method: results from a general population study. Health Econ..

[13] NICE (National Institute for Health and Care Excellence) (2013). Process and methods guides: guide to the methods of technology appraisal 2013.

[14] Konig HH, Gunther OH, Angermeyer MC, Roick C (2009). Utility assessment in patients with mental disorders: validity and discriminative ability of the time trade-off method. Pharmacoeconomics.

[15] Osborne RH, De Abreu Lourenco R, Dalton A, Houltram J, Dowton D, Joshua DE, Lindeman R, Ho PJ (2007). Quality of life related to oral versus subcutaneous iron chelation: a time trade-off study. Value Health.

[16] Arnesen T, Trommald M (2005). Are QALYs based on time trade-off comparable? A systematic review of TTO methodologies. Health Econ..

[17] Matza LS, Chung K, Van Brunt K, Brazier JE, Braun A, Currie B, Palsgrove A, Davies E, Body JJ (2013). Health state utilities for skeletal-related events secondary to bone metastases. Eur. J. Health Econ..

[18] Stevenson LW, Hellkamp AS, Leier CV, Sopko G, Koelling T, Warnica JW, Abraham WT, Kasper EK, Rogers JG, Califf RM (2008). Changing preferences for survival after hospitalization with advanced heart failure. J. Am. Coll. Cardiol..

[19] Bryce CL, Angus DC, Switala J, Roberts MS, Tsevat J (2004). Health status versus utilities of patients with end-stage liver disease. Qual. Life Res..

[20] Eldabe S, Lloyd A, Verdian L, Meguro M, Maclaine G, Dewilde S (2010). Eliciting health state utilities from the general public for severe chronic pain. Eur. J. Health Econ..

[21] Wilke DR, Krahn M, Tomlinson G, Bezjak A, Rutledge R, Warde P (2010). Sex or survival: short-term versus long-term androgen deprivation in patients with locally advanced prostate cancer treated with radiotherapy. Cancer.

[22] Bozic KJ, Chiu VW, Slover JD, Immerman I, Kahn JG (2011). Health state utility in patients with osteoarthritis of the hip and total hip arthroplasty. J. Arthroplast..

[23] Hsu PC, Federico CA, Krajden M, Yoshida EM, Bremner KE, Anderson FH, Weiss AA, Krahn MD (2012). Health utilities and psychometric quality of life in patients with early- and late-stage hepatitis C virus infection. J. Gastroenterol. Hepatol..

[24] Hsu PC, Krajden M, Yoshida EM, Anderson FH, Tomlinson GA, Krahn MD (2009). Does cirrhosis affect quality of life in hepatitis C virus-infected patients?. Liver Int..

[25] Ram AN, Curtin CM, Chung KC (2009). Population-based utilities for upper extremity functions in the setting of tetraplegia. J. Hand Surg. Am..

[26] Cavaliere CM, Chung KC (2010). A cost-utility analysis of nonsurgical management, total wrist arthroplasty, and total wrist arthrodesis in rheumatoid arthritis. J. Hand Surg. Am..

[27] Havrilesky LJ, Broadwater G, Davis DM, Nolte KC, Barnett JC, Myers ER, Kulasingam S (2009). Determination of quality of life-related utilities for health states relevant to ovarian cancer diagnosis and treatment. Gynecol. Oncol..

[28] Levy AR, Christensen TL, Johnson JA (2008). Utility values for symptomatic non-severe hypoglycaemia elicited from persons with and without diabetes in Canada and the United Kingdom. Health Qual. Life Outcomes.

[29] Sinno HH, Thibaudeau S, Duggal A, Lessard L (2010). Utility scores for facial disfigurement requiring facial transplantation [outcomes article]. Plast. Reconstr. Surg..

[30] Trent M, Lehmann HP, Qian Q, Thompson CB, Ellen JM, Frick KD (2011). Adolescent and parental utilities for the health states associated with pelvic inflammatory disease. Sex Transm. Infect..

[31] Bejia I, Salem KB, Touzi M, Bergaoui N (2006). Measuring utilities by the time trade-off method in Tunisian rheumatoid arthritis patients. Clin. Rheumatol..

[32] Bossema E, Stiggelbout A, Baas-Thijssen M, van de Velde C, Marijnen C (2008). Patients’ preferences for low rectal cancer surgery. Eur. J. Surg. Oncol..

[33] Chen CL, Kuppermann M, Caughey AB, Zane LT (2008). A community-based study of acne-related health preferences in adolescents. Arch. Dermatol..

[34] Guest JF, Naik N, Sladkevicius E, Coombs J, Gray EJ (2012). Utility values for chronic myelogenous leukemia chronic phase health states from the general public in the United Kingdom. Leuk. Lymphoma.

[35] Khanna D, Ahmed M, Furst DE, Ginsburg SS, Park GS, Hornung R, Tsevat J (2007). Health values of patients with systemic sclerosis. Arthritis Rheum..

[36] Mennini FS, Panatto D, Marcellusi A, Cristoforoni P, De Vincenzo R, Di Capua E, Ferrandina G, Petrillo M, Sasso T, Ricci C (2011). Time trade-off procedure for measuring health utilities loss with human papillomavirus-induced diseases: a multicenter, retrospective, observational pilot study in Italy. Clin. Ther..

[37] Saw SM, Gazzard G, Gomezperalta C, Au Eong KG, Seah S (2005). Utility assessment among cataract surgery patients. J. Cataract. Refract. Surg..

[38] Schmitt J, Meurer M, Klon M, Frick KD (2008). Assessment of health state utilities of controlled and uncontrolled psoriasis and atopic eczema: a population-based study. Br. J. Dermatol..

[39] Heintz E, Krol M, Levin LA (2013). The impact of patients’ subjective life expectancy on time tradeoff valuations. Med. Decis. Mak..

[40] Attema AE, Brouwer WB (2010). On the (not so) constant proportional trade-off in TTO. Qual. Life Res..

[41] Lin MR, Yu WY, Wang SC (2012). Examination of assumptions in using time tradeoff and standard gamble utilities in individuals with spinal cord injury. Arch. Phys. Med. Rehabil..

[42] Stiggelbout AM, Kiebert GM, Kievit J, Leer JW, Habbema JD, De Haes JC (1995). The “utility” of the time trade-off method in cancer patients: feasibility and proportional trade-off. J. Clin. Epidemiol..

[43] Rowen D, Brazier J, Glied S, Smith PC (2011). Health Utility Measurement. The Oxford Handbook of Health Economics.

[44] Torrance GW (1986). Measurement of health state utilities for economic appraisal. J. Health Econ..

[45] Brazier J (2008). Valuing health States for use in cost-effectiveness analysis. Pharmacoeconomics.

[46] PBAC (Pharmaceutical Benefits Advisory Committee) (2008). Guidelines for preparing submissions to PBAC, Version 4.3.2.

[47] Feeny D, Blanchard CM, Mahon JL, Bourne R, Rorabeck C, Stitt L, Webster-Bogaert S (2004). The stability of utility scores: test-retest reliability and the interpretation of utility scores in elective total hip arthroplasty. Qual. Life Res..

[48] Laupacis A, Bourne R, Rorabeck C, Feeny D, Wong C, Tugwell P, Leslie K, Bullas R (1993). The effect of elective total hip replacement on health-related quality of life. J. Bone Jt. Surg. Am..

[49] van Nooten FE, Koolman X, Busschbach JJ, Brouwer WB (2013). Thirty down, only ten to go?! awareness and influence of a 10-year time frame in TTO. Qual. Life Res..

[50] Feeny D, Fayers P, Hays R (2005). Preference-based measures: utility and quality-adjusted life years. Assessing quality of life in clinical trials.

[51] Scalone, L., Stalmeier, P.F., Milani, S., Krabbe, P.F.: Values for health states with different life durations. Eur. J. Health Econ. (2014). doi:10.1007/s10198-014-0634-0 10.1007/s10198-014-0634-0 25266301

[52] Feeny D (2013). Standardization and regulatory guidelines may inhibit science and reduce the usefulness of analyses based on the application of preference-based measures for policy decisions. Med. Decis. Mak..

